# Effects of Additives on Electrochromic Properties of Nanocrystalline Tungsten Oxide Films Prepared by Complexation-Assisted Sol–Gel Method

**DOI:** 10.3390/ma16072681

**Published:** 2023-03-28

**Authors:** Dan Zhou, Zhibo Tong, Hongmei Xie, Jiaotong Sun, Fenggui Chen

**Affiliations:** Chongqing Key Laboratory of Extraordinary Bond Engineering and Advanced Materials Technology, College of Materials Science and Engineering, Yangtze Normal University, Chongqing 408100, China

**Keywords:** tungsten oxide, nanocrystalline, electrochromic property, additive, complexation, sol–gel method, film

## Abstract

To improve the electrochromic (EC) properties of sol–gel-derived WO_3_ films, a series of organic small molecules, such as dopamine (DA), catechol, tyramine, phenol and 2-phenylethylamine, were added into peroxotungstic acid precursor sols as structure-directing additives, and five modified WO_3_ films were prepared by a simple and low-cost complexation-assisted sol–gel method. The effects of the above additives on the EC properties of the modified WO_3_ films have been studied in detail. Compared with the pure WO_3_ polycrystalline film, all the modified films combine the advantages of nanocrystalline and amorphous phases and show higher EC properties attributed to the unique nanocrystal-embedded amorphous structure. The results indicate that different additives with different numbers and types of functional groups (hydroxyl and amino groups) can change the microstructure, morphology, and thus electrochemical and EC properties of the films in various degrees. The additives, in order of their strong interactions with the sols, are DA, catechol, tyramine, phenol and 2-phenylethylamine, primarily depending on the number of hydroxyl groups. Of all the additives, DA with both catechol hydroxyl and amino groups shows the most positive effect; that is, the WO_3_ film modified with DA exhibits the best EC properties in terms of contrast, switching speed, stability, and coloration efficiency.

## 1. Introduction

In recent years, under the background of sustainable development, the electrochromic (EC) smart window has been considered as a promising energy-conservation and emission-reduction device for green buildings, vehicles, etc. and received more and more attention [1,2]. EC materials, the most important component of a smart window, can change their optical properties (absorption, reflectance, transmittance, etc.) reversibly under a small applied voltage, and they have also been widely applied to energy-saving displays and antiglare automobile rear-view mirrors, in addition to smart windows, which are thought to be one of the most potential green energy-saving materials [3,4,5].

Transition metal oxides are commonly used as EC materials [3,5,6]. Among them, tungsten oxide (WO_3_) is the most studied and extremely attractive one due to its high transmittance contrast, thermal and photo stability, good ionic and electronic conductivity, and improved lifetime of the devices. Many techniques can be used to prepare WO_3_ EC films, including chemical vapor deposition (CVD) [7], sputtering [8], evaporation [9] and so on. However, the WO_3_ films produced by the above methods are expensive and inefficient because of the special equipment and high vacuum conditions, impeding their commercial applications. Therefore, the sol–gel method [10,11,12], a wet-chemical route, is attractive and regarded as an alternative owing to its low cost, simplicity, repeatable process, and uniform and large-area film formation. Moreover, during the sol–gel process, the microstructure, crystal size, porosity and composition of the deposited films can be easily controlled by optimizing various preparation parameters or introducing additives into the sols [13], which will affect the kinetics, durability and coloration efficiency of the thin films [14].

The EC performances of the WO_3_ films prepared by the traditional sol–gel method, however, are still not good enough for actual use, such as inadequate optical contrast, switching time, long-term electrochemical cycling stability or coloration efficiency. Many researchers have tried to adjust and control the structures/morphology of the sol–gel-derived WO_3_ films by using organic additives to improve their EC performances. Research works can be mainly divided into two categories: one is to add pore-forming templates (polymers or surfactants) into the sol and then remove the templates through heat treatment or solvent extraction [15,16,17,18]. The obtained high-porosity WO_3_ films possess a very large specific surface area, which can increase the color response speed and optical contrast. However, the stability of the high-porosity structure decreases [19], because part of the structure is easily destroyed or collapsed during the heat treatment process or the surface erosion rate is accelerated during the electrochemical cycling test. The other is to add a large amount of alcohols, organic acids or other organic compounds into the precursor sols to increase the contrast and switching speed [20,21]. Unfortunately, this kind of method cannot effectively enhance the combination property of the films and may bring some environmental and health problems. Therefore, it is highly desirable to develop sol–gel-derived WO_3_ EC films with excellent optical contrast, switching time, cycling stability, and coloration efficiency satisfying practical requirements.

It has been demonstrated that the EC properties of the films are greatly affected by the structure and morphology. It is well known that an amorphous WO_3_ film is characterized by a short switching time, high coloration efficiency and high contrast due to its loose structure and large surface area [13,18,21]. However, because an amorphous film has many structural defects, the electrochemical properties of the film will irreversible deteriorate during the EC cycling. On the other hand, a highly crystalline WO_3_ film shows high electrochemical stability owing to its compact and ordered structure, but it may be too dense for ions diffusion and intercalation, leading to a slow response time or low contrast. A number of efforts have been undertaken to overcome the problems and combine the advantages of the two structures in nanoscale [22].

In our previous work, a complexation-assisted sol–gel method was proposed in order to improve the EC properties of traditional sol–gel-derived WO_3_ films, in which a small amount of dopamine (DA) was added into peroxotungstic acid (PTA) precursor sol to produce PTA–DA complex sol [23]. The DA molecules suppressed the formation of very large WO_3_ particles in the precursor sol and inhibited the formation of a highly stressed polycrystalline structure during annealing. The DA-modified WO_3_ films exhibited much higher optical contrast, coloration efficiency, switching speed, and excellent long-term cycling stability (up to 35,000 cycles) than the pure WO_3_ film due to nanocrystal surrounded by amorphous matrix.

So far, however, very little work has been completed to investigate the function of different additives or complexing agents during the preparation and application of the sol–gel-derived WO_3_ films in detail. In this paper, therefore, a series of organic small molecules, such as DA, catechol (CA), tyramine (TA), phenol (Ph) and 2-phenylethylamine (PEA), are added into PTA precursor sol as structure-directing additives, and a variety of modified WO_3_ films are prepared by a simple and low-cost complexation-assisted sol–gel method to study the effects of the above additives containing different numbers and types of functional groups (hydroxyl and amino groups) on the structure, morphology, electrochemical and EC properties of the modified WO_3_ films.

## 2. Materials and Methods

### 2.1. Materials

DA hydrochloride (98%), CA (99%), TA hydrochloride (98%), Ph (≥99%), PEA hydrochloride (98%), 30% H_2_O_2_, lithium perchlorate (LiClO_4_) (99.9%), propylene carbonate (PC) (≥98%), acetone (98%) and isopropanol (99%) were obtained from Shanghai Aladdin Chemical Reagent Co., Ltd. (Shanghai, China). Tungsten powder (≥99%) was received from Merck KGaA (Darmstadt, Germany). Conducting indium tin oxide-coated glass (ITO glass, <7 Ω/□) was purchased from Zhuhai Kaivo Optoelectronic Technologies Co. (Zhuhai, China).

### 2.2. Preparation of the Modified WO_3_ Films

Prior to use, the ITO glass was cleaned with detergent, deionized H_2_O, acetone, and isopropanol for 10 min in an ultrasonic bath, respectively. Then, the ITO glass was further cleaned by a plasma treatment for 5 min to improve their hydrophilicity.

The modified WO_3_ films were produced through our previous complexation-assisted sol–gel method [23] with slight modification. First, 20 mmol tungsten powder was added in 60 mL 30% H_2_O_2_ solution at 0 °C under continuously magnetic stirring to form a colorless transparent PTA, which was followed by a reflux at 60 °C for 6 h to decompose of excess peroxide. After slow stirring for one week, 3.3 mmol DA was introduced into the PTA sol to obtain concentrated yellow PTA/DA complex sol after heating at 60 °C for 10 h. The PTA/DA sol was coated on the cleaned ITO glass by spin-coating technology, and the as-prepared film was dried at 60 °C for 1 h followed by annealing at 300 °C for 2 h to form the modified WO_3_/DA film. The film thickness was controlled at about 200 nm. Meanwhile, other modified WO_3_ films by adding CA, TA, Ph, and PEA were prepared in the same way, respectively. The as-prepared films are denoted as WO_3_/DA-300 °C, WO_3_/CA-300 °C, WO_3_/TA-300 °C, WO_3_/Ph-300 °C, and WO_3_/PEA-300 °C, respectively. For comparison, the pure WO_3_-300 °C film was also prepared under the similar conditions except in the absence of additives.

For Fourier transform infrared spectroscopy (FTIR) characterization, xerogel samples were obtained by drying the PTA and PTA/additive complex sols in vacuum oven at 60 °C for 24 h and marked as WO_3_, WO_3_/DA, WO_3_/CA, WO_3_/TA, WO_3_/Ph and WO_3_/PEA xerogels, respectively.

### 2.3. Characterization of the Modified WO_3_ Films

The UV-Vis absorption spectra (400–800 nm) of various sols and films were obtained on a UV-VIS-NIR spectrophotometer (Shimadzu UV-3600, Tokyo, Japan). The FTIR spectra of the xerogels and films from 400 to 4000 cm^−1^ were recorded on a Nicolet is5 FTIR spectrometer (Thermo Fisher Scientific, Waltham, MA, USA). The morphology and microstructure of the films were observed by a field-emission scanning electron microscope (FESEM, ZEISS GeminiSEM 300, Oberkochen, Germany) and a transmission electron microscope (TEM, JEOL-2100F, Tokyo, Japan). X-ray diffraction (XRD) analyses of all the samples were characterized by a Shimadzu XRD-6100 diffractometer with an X-ray wavelength of 1.542 Å (Cu Kα radiation, 40 kV and 30 mA) over a 2-theta range of 10–60°.

### 2.4. Measurement of Electrochemical and EC Properties

The electrochemical properties of the films were tested using a three-electrode system with LiClO_4_/PC (1 mol/L) as a electrolyte, Pt sheet as a counter-electrode, Ag/AgCl as a reference electrode, and the prepared films coated on ITO glass as working electrodes. Nyquist plots of the films were performed using an AUTOLAB PGSTAT 302N potentiostat/galvanostat analyzer (Eco Chemie) at a perturbation voltage of 10 mV in the frequency range of 10 kHz to 10 mHz. Cyclic voltammetry (CV) plots were collected on an AUTOLAB analyzer at a scan rate of 100 mV s^−1^ from −1.0 to +1.0 V, and all current densities were normalized to the geometric surface area of the electrodes.

EC properties (visible spectra (400–800 nm) and dynamic switching curves at 720 nm) of the films were recorded on a Shimadzu UV-3600 spectrophotometer in combination with an AUTOLAB analyzer by applying constant potentials and square-wave potentials (oscillating between +1.0 V and −1.0 V at a time step of 50 s), respectively.

## 3. Results and Discussion

### 3.1. Preparation and Characterization of the Modified WO_3_ Films

**Figure 1:**
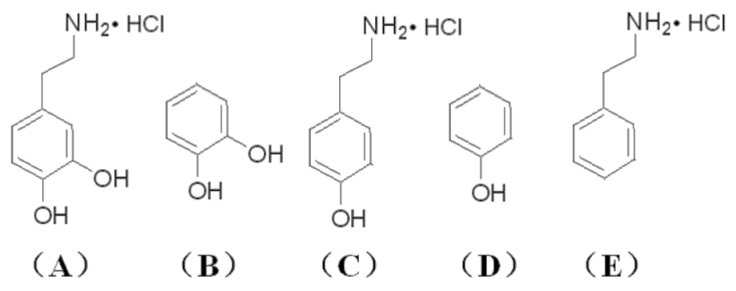
Structural formulas of (**A**) DA hydrochloride, (**B**) CA, (**C**) TA hydrochloride, (**D**) Ph and (**E**) PEA hydrochloride.

In order to study the effects of the additives, that is, different numbers and types of functional groups (hydroxyl and amino groups), on the structure, morphology, electrochemical and EC properties of WO_3_ films prepared by the sol–gel method, DA, CA, TA, Ph, and PEA were selected as additives and added into the PTA precursor sols to produce a variety of modified WO_3_ films. Figure 1 displays the structural formulas of these five additives or their hydrochlorides. DA contains both catechol hydroxyl and amino functional groups in which the catechol units are well known to show quite strong affinity to various kinds of metals/metal ions [24], and the amino groups are mainly in the form of ammonium salt. The catechol hydroxyl groups of DA can bind uncoordinated tungsten atoms on the surface of PTA colloid nanoparticles strongly via the deprotonated O, O’ sites [25,26,27] in monodentate, bidentate, or chelated modes [28]. There is also the possibility of hydrogen bonding between the hydroxyl groups on DA and O atoms on PTA. CA has catechol hydroxyl groups and no amino group. TA possesses one hydroxyl group and one amino group. Meanwhile, both Ph and PEA are monofunctional compounds, containing only one hydroxyl group and one amino group, respectively.

**Figure 2:**
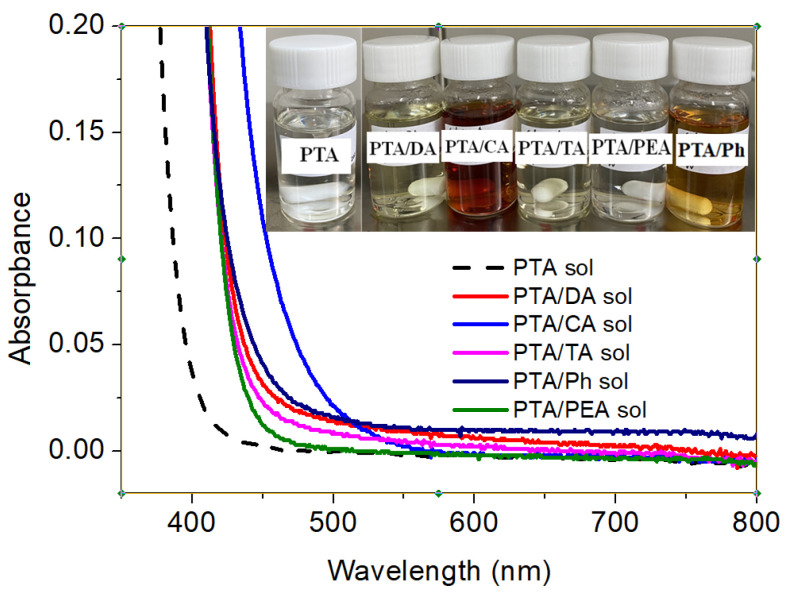
UV-Vis absorption spectra of the PTA, PTA/DA, PTA/CA, PTA/TA, PTA/Ph, and PTA/PEA sols. The inset showing the photos of these sols.

A nearly colorless and transparent PTA sol was formed after one-week stirring of the reactive mixture of tungsten powder and H_2_O_2_, as shown in the photo of the inset of Figure 2. When the additives were introduced into the sols, respectively, the color of the complex sols immediately darkened significantly (inset of Figure 2), indicating an instantaneous formation of charge-transfer complexes due to the strong complexation, hydrogen bonding, or electrostatic interaction between the functional groups of the additives and tungsten or oxygen atoms of PTA colloidal nanoparticles. Furthermore, as can be seen from Figure 2, UV-Vis absorption spectra of these complex sols show a red-shift and enhanced intensity compared with that of the pure PTA sol, which is similar to the phenomenon observed from DA-TiO_2_ complexes [29,30].

**Figure 3:**
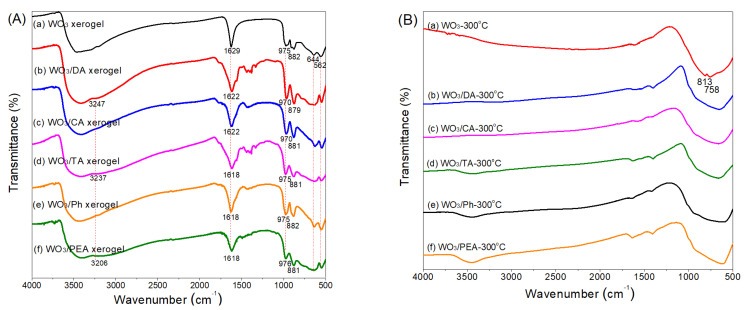
(**A**) FTIR spectra of (a) WO_3_, (b) WO_3_/DA, (c) WO_3_/CA, (d) WO_3_/TA, (e) WO_3_/Ph and (f) WO_3_/PEA xerogels, and (**B**) FTIR spectra of (a) WO_3_-300 °C, (b) WO_3_/DA-300 °C, (c) WO_3_/CA-300 °C, (d) WO_3_/TA-300 °C, (e) WO_3_/Ph-300 °C and (f) WO_3_/PEA-300 °C films.

To prove the successful preparation of the complex sols and study the interactions between the PTA sol and the additives, all the PTA and complex sols were dried in a vacuum oven at 60 °C for 24 h to form xerogels, and the FTIR spectra of the xerogels are compared in Figure 3A. As can be seen from Curve a of Figure 3A, the broad absorption band of the pure WO_3_ xerogel at wavenumbers around 3500 cm^−1^ is caused by the stretching vibration υ(OH) of water and hydroxyl adsorbed or incorporated in the xerogel, and the peak at 1629 cm^−1^ can be attributed to the in-plane bending vibration δ(HOH) of water molecules [31]. In addition, the pure WO_3_ xerogel shows key characteristic absorption bands of amorphous WO_3_, for example, the peaks corresponding to the stretching vibration of terminal W=O bonds (975 cm^−1^) on the surface of the nanoparticles and micro-voids of the xerosol [32], the stretching vibration of bridging peroxide W–O–O–W (882 cm^−1^), the W–O–W bridging mode of the W–O_6_ corner-sharing octahedral (644 cm^−1^), and the W−O deformation mode (562 cm^−1^) [10,31,32,33]. After addition of the additives, besides those from WO_3_, some new bands appear, such as a series of weak bands at 1100–1600 cm^−1^ attributed to the N−H, C−H, C−N, C−C, and aromatic C−C bonds due to the additives, as shown in Curve b–f. In addition, broad and strong bands at 3247, 3237, and 3206 cm^−1^ occur corresponding to the stretching vibration of N−H of DA, TA and PEA for Curve b, d and f, respectively. DA, TA and PEA all contain an amino group but different numbers of hydroxyl groups. The DA molecule has two adjacent hydroxyl groups that can preferentially bind tungsten strongly via the deprotonated O, O′ sites and the interaction between the amino group and PTA may be very limited. The TA molecule has only one hydroxyl group to link tungsten, and the PEA molecule has no hydroxyl group and thus may only form hydrogen bonding or an electrostatic interaction between NH_2_ or NH_3_^+^ groups on PEA and O atoms on the xerogel, which consequently causes these stretching vibration bands of N−H to shift to lower frequencies. Therefore, it is inferred that the interaction order, in which the additives are preferentially bonded with the xerogel, may be the catechol hydroxyl groups, hydroxyl group, and aliphatic side chain (NH_2_ or NH_3_^+^). Certainly, these newly emerging peaks imply the coexistence of tungsten oxide and organic additives. The superposition of the bending vibration of water molecules in WO_3_ xerogel and O−H and/or N−H bonds in complex xerogels makes the peaks stronger and wider, which shift to lower wavenumbers (from 1629 cm^−1^ to about 1620 cm^−1^) owing to the interaction of the additives and PTA. Moreover, after the addition of DA and CA into the PTA sol, both the terminal W=O peaks of these two complex xerogels move in the lower wavenumber direction (from 975 cm^−1^ for the WO_3_ xerogel to 970 cm^−1^ for WO_3_/DA and WO_3_/CA serogels) because the catechol hydroxyl groups of DA and CA are bidentate, showing a relatively strong complexation with W atoms, while the W=O peak positions of other complex xerogels containing monodentate change little. The peaks of the WO_3_ complex xerogels corresponding to the stretching vibrations of W–O–W bands and the W−O deformation mode downshift obviously from 644 cm^−1^ to about 630 cm^−1^ and from 562 cm^−1^ to about 550 cm^−1^, respectively, further verifying the formation of the complexes by the interactions between phenolic hydroxyl groups or amino groups (NH_2_ or NH_3_^+^) of the additives and W or O species of PTA. In addition, oxidation products of the additives containing phenolic hydroxyl groups (DA, CA, TA and Ph), such as quinone, were also found according to the weak bands at about 1700–1770 cm^−1^, meaning some of the phenolic hydroxyl groups were also oxidized slightly during the complexation process.

After spin-coating of all the PTA and complex sols and then annealing at 300 °C for 2 h, the obvious decrease in the intensity or even disappearance of W=O and W−O−O−W absorption bands of the WO_3_-300 °C and modified films (Curve a-f in Figure 3B) can be attributed to the aging and condensation of WO_6_ octahedral units at their corners to form a W–O–W network [33]. Furthermore, the loss of structural and absorbed water when heating at 300 °C results in the weakening of the bands at ~3500 cm^−1^ and ~1630 cm^−1^. The absorbance bands at ~1400 cm^−1^ can be ascribed to the W–OH bending vibration. At the same time, the weak bands attributed to the additives at 1100–1600 cm^−1^ disappear due to the burning off organic additives after annealing at 300 °C for 2 h. On the other hand, the intensity increase in the W–O–W band and well-defined double bands at 813 cm^−1^ and 758 cm^−1^ for the WO_3_-300 °C film (Curve a in Figure 3B) reveal the crystalline structure of WO_3_. In contrast, the absence of the double bands and the obvious broad bands at 600–670 cm^−1^ in Curve b–f indicate that the dominant structure of the films modified by the additives is different from crystalline one of the WO_3_-300 °C film.

The structures and morphologies of the WO_3_ films were studied by electron microscopes. Figure S1 shows FESEM images of the WO_3_-300 °C and modified films, revealing that all the films present relatively smooth, uniform and close-packed surfaces.

**Figure 4:**
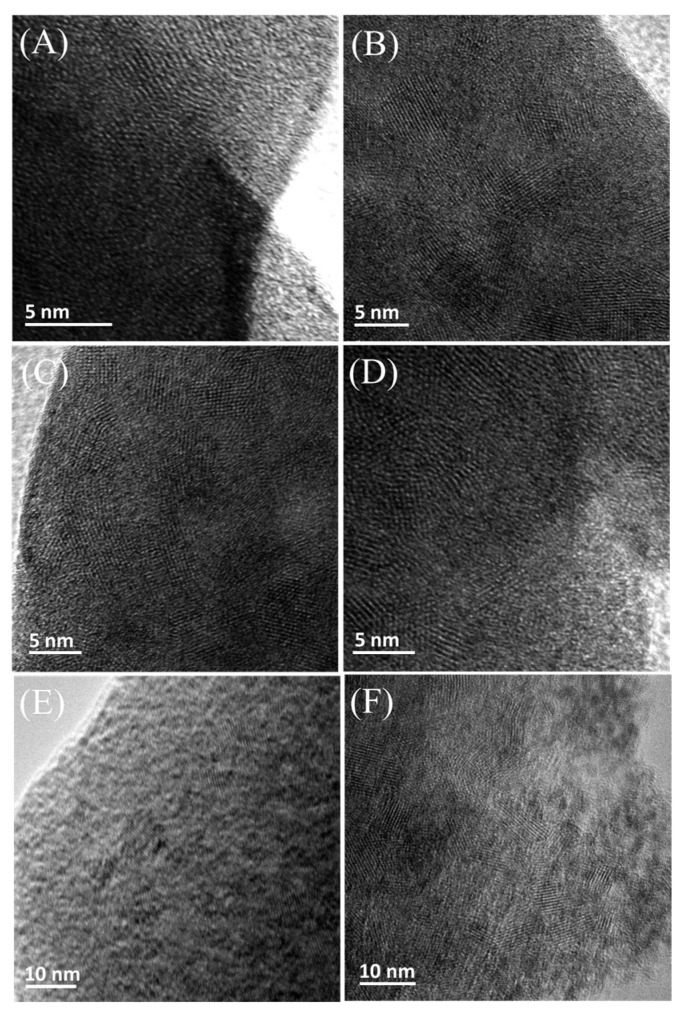
TEM images of (**A**) WO_3_-300 °C, (**B**) WO_3_/DA-300 °C, (**C**) WO_3_/CA-300 °C, (**D**) WO_3_/TA-300 °C, (**E**) WO_3_/Ph-300 °C, and (**F**) WO_3_/PEA-300 °C films.

The TEM images of all the films can be seem from Figure 4. Different from the WO_3_-300 °C film exhibiting uniform polycrystalline structure (Figure 4A), the modified films show that WO_3_ nanocrystals are uniformly separated from each other and distributed in an amorphous WO_3_ matrix (Figure 4B–F). The amorphous matrix surrounding the WO_3_ nanocrystals may act as ion transport channels and increase reactive active sites during the EC process, which is beneficial for the diffusion of the electrolyte into the nanocrystals [10] and the improvement of the optical contrast and switching response. Meanwhile, the amorphous phase in the nanocrystal-embedded films may also serve as buffer layers to accommodate the volume change produced by ion insertion/extraction [18] and avoid bond breakage during electrochemical cycling. The amorphous and nanocrystalline regions were uniformly distributed, which may decrease the stress concentration and inhibit erosion produced by high local stress, contributing to high cycling stability [23]. According to Figure 4B–F, the estimated particle sizes of the crystalline grains increase gradually in the range of from about 5 to 20 nm in the following order: WO_3_/DA-300 °C, WO_3_/CA-300 °C, WO_3_/TA-300 °C, WO_3_/Ph-300 °C and WO_3_/PEA-300 °C. Due to the high coordination ability of catechol hydroxyl groups on metals, the strong complexation of catechol additives (such as DA and CA) can limit the size of colloidal particles and destroy the structure order of particles to a certain extent, which results in the formation of more uniform and finer nanocrystals distributed in an amorphous matrix after heat treatment at 300 °C. Compared with CA, DA also contains NH_3_^+^, which can help PTA colloidal particles to better disperse in colloid and thus stabilize colloid solution. However, the complexation ability of monodentate additives (TA, Ph and PEA) is slightly poor, especially for PEA containing only one amino group without hydroxyl group, resulting in the formation of relatively large and heterogeneous nanocrystals after annealing. Different numbers and types of functional groups in the additives may have different effects on the microstructure and morphology of the films, therefore, and will ultimately result in a large difference in the properties of the films including EC property. Moreover, according to TEM images, clear lattice fringes of nanocrystals can be seen for the WO_3_-300 °C and modified films, and the lattice spacing values (3.83, 3.77, and 3.66 Å) perfectly fit monoclinic WO_3_ (JCPDS 43-1035), corresponding to the d-spacing of (002), (020), and (200) planes.

**Figure 5:**
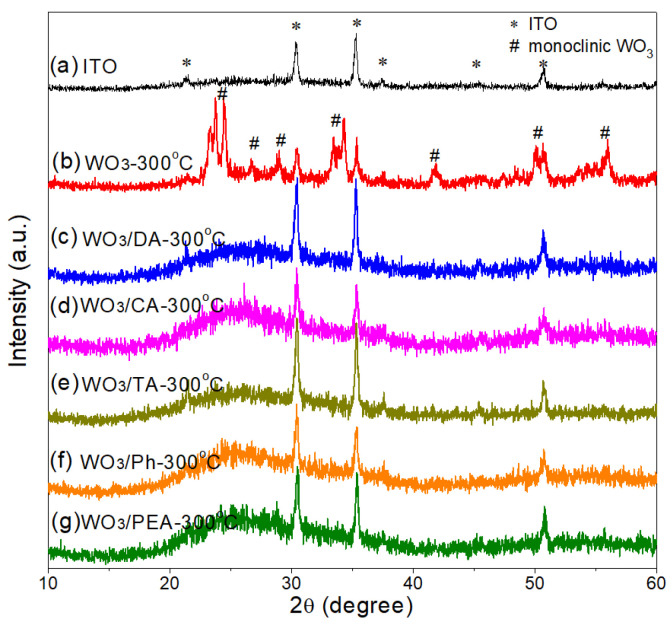
X-ray diffraction (XRD) patterns of (a) ITO glass, (b) WO_3_-300 °C, (c) WO_3_/DA-300 °C, (d) WO_3_/CA-300 °C, (e) WO_3_/TA-300 °C, (f) WO_3_/Ph-300 °C, and (g) WO_3_/PEA-300 °C films.

Figure 5 shows XRD patterns of ITO glass, WO_3_-300 °C, WO_3_/DA-300 °C, WO_3_/CA-300 °C, WO_3_/TA-300 °C, WO_3_/Ph-300 °C, and WO_3_/PEA-300 °C films coated on ITO glass. After annealing at 300 °C for 2 h, the WO_3_-300 °C film displays several relatively intense and distinct diffraction peaks at 2θ = 23.2, 23.7, 24.4 and 34.2° in Curve b corresponding to (002), (020), (200) and (202) planes, respectively, indexed to the monoclinic crystalline WO_3_ (JCPDS Card No.43-1035). However, for the modified films (Curve c-g), only weak and broad humps appear in the 2θ region from 20° to 30° in addition to several characteristic peaks of the ITO substrate, as shown in Curve a. During the preparation of the modified films, the additives may inhibit crystal growth, causing the generation of the small WO_3_ nanocrystals embedded in an amorphous matrix, which is in agreement with the FTIR and TEM results, which broaden and weaken the diffraction peaks. Therefore, it is difficult to observe distinct diffraction peaks [30,34].

### 3.2. Electrochemical Properties of the Modified WO_3_ Films

**Figure 6:**
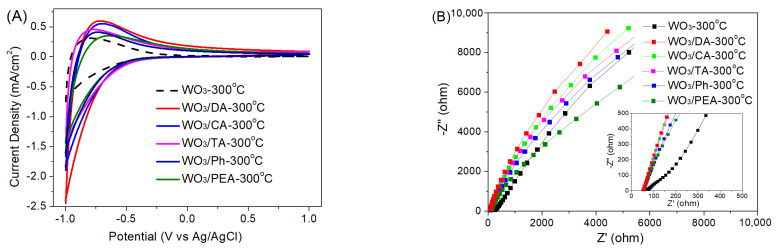
(**A**) CV curves tested at a scan rate of 100 mV s^−1^ between −1.0 and +1.0 V and (**B**) Nyquist plots of the WO_3_-300 °C, WO_3_/DA-300 °C, WO_3_/CA-300 °C, WO_3_/TA-300 °C, WO_3_/Ph-300 °C and WO_3_/PEA-300 °C films in 1 mol/L LiClO_4_/PC at frequency from 100 kHz to 10 mHz using a perturbation amplitude of 10 mV at the potential of 0 V.

The electrochemical properties of the films during ion insertion/extraction were studied by using CV. Figure 6A displays the CV curves of the WO_3_-300 °C and modified WO_3_ films tested at a scan rate of 100 mV s^−1^ between −1.0 and +1.0 V. For all the films, the CV curves are similar in shape, but the modified films exhibit a higher peak current density and CV integral area than the WO_3_-300 °C film, and the order of the modified films from high to low is roughly WO_3_/DA-300 °C, WO_3_/CA-300 °C, WO_3_/TA-300 °C, WO_3_/Ph-300 °C and WO_3_/PEA-300 °C. Therefore, the modified films have more electrochemical active mass and higher ion/electron storage capacity, that is, more ions/electrons are involved at the interface between the films and electrolyte than the WO_3_-300 °C film, and thus, it is deduced that the modified films will show better EC performances due to the nanocrystal-embedded amorphous structure.

Electrochemical impedance spectroscopy (EIS) was applied to further investigate the electrochemical behaviors of the modified WO_3_ films and provided information on the interfaces of charge transfer and ion diffusion. The Nyquist plots of the WO_3_-300 °C and modified WO_3_ films can be seen in Figure 6B. Compared with the WO_3_-300 °C, the modified WO_3_ films (especially WO_3_/DA-300 °C) exhibit smaller intercepts of the curves at the *X*-axis and depressed arc diameters in the high-frequency region (the inset of Figure 6B) and higher slopes in the low-frequency region (except WO_3_/PEA-300 °C), meaning lower series resistance and charge-transfer resistance and a higher diffusion rate of Li^+^ ion for ion insertion into and extraction from the films [35,36,37]. The results indicate that the nanocrystal-embedded amorphous phase structure of the modified films is more beneficial to both Li^+^ ion diffusion and charge transfer compared with the compact polycrystalline structure of WO_3_-300 °C.

### 3.3. EC properties of the Modified WO_3_ Films

**Figure 7:**
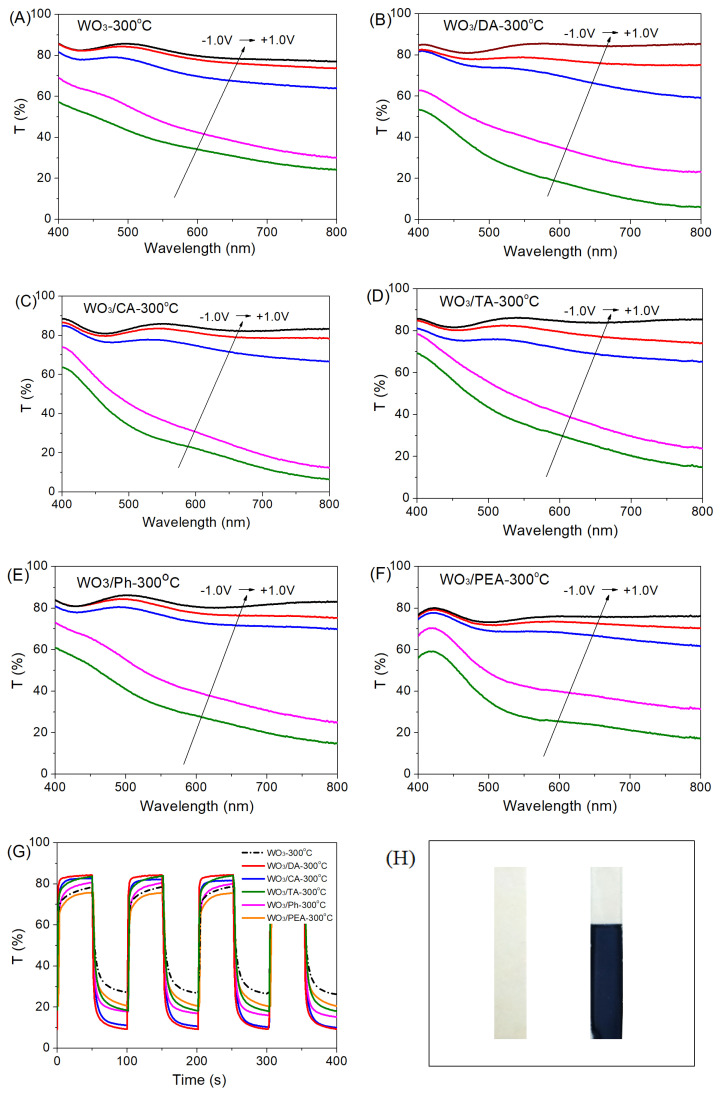
UV-vis transmittance spectra at different potentials of (**A**) pure WO_3_-300 °C, (**B**) WO_3_/DA-300 °C, (**C**) WO_3_/CA-300 °C, (**D**) WO_3_/TA-300 °C, (**E**) WO_3_/Ph-300 °C, and (**F**) WO_3_/PEA-300 °C films at the potentials of −1.0, −0.8, +0.5, +0.8 and +1.0 V, as shown in green, violet, blue, red and black lines, respectively, (**G**) switching curve comparison between the WO_3_-300 °C, WO_3_/DA-300 °C, WO_3_/CA-300 °C, WO_3_/TA-300 °C, WO_3_/Ph-300 °C, and WO_3_/PEA-300 °C films at λ_720nm_ (+1.0 V/−1.0 V, 100 s per cycle), and (**H**) the photo of the WO_3_/DA-300 °C films in the bleached and colored states respectively.

The EC process of WO_3_ films involves the double injection/extraction of cations and electrons into/from the films and the color switches between blue and transparent. EC properties of the pure WO_3_-300 °C and modified films were tested and compared as shown in Figure 7 and Table 1. Transmittance contrast or modulation, Δ*T*, is defined as the difference in the transmittance between the bleached and the colored states at a certain wavelength. According to UV-vis transmittance spectra (Figure 7A–F), ∆T values of the WO_3_-300 °C, WO_3_/DA-300 °C, WO_3_/CA-300 °C, WO_3_/TA-300 °C, WO_3_/Ph-300 °C and WO_3_/PEA-300 °C films at a wavelength of 720 nm are about 51, 75, 72, 66, 64, and 56%, respectively, as listed in Table 1. Compared with the pure WO_3_-300 °C film, all the modified WO_3_ films possess much higher optical contrasts (∆*T*) because the nanocrystal-embedded amorphous structure produced by the additives can provide more active sites and higher ion/electron storage capacity than the compact polycrystalline structure. Therefore, as we can see from the aforementioned test results related to the characterization and electrochemical properties, different numbers and types of functional groups in the additives may ultimately result in a large difference in the EC properties of the films by changing the microstructure and morphology of the films. In other words, the addition of the additives with more hydroxyl groups produces the modified WO_3_ films with better performances.

Coloration efficiency (*CE*) is defined as the change in optical density (Δ*OD*) measured at a certain wavelength (*λ*) per unit charge (*Q*) inserted into or extracted from the EC films, namely, *CE* = Δ*OD*(*λ*)/(*Q*/*A*) = log(*T_b_*/*T_c_*)/*q* [38], where *q* is the charge density (injected/ejected charge *Q* per unit electrode area *A*), and *T_b_* and *T_c_* represent the transmittances in the bleached and colored states. Coloration efficiency is also an important characteristic parameter for evaluating EC performance. The coloration efficiency values were calculated to be 66, 90, 85, 81, 77 and 70 cm^2^ C^−1^ for the WO_3_-300 °C, WO_3_/DA-300 °C, WO_3_/CA-300 °C, WO_3_/TA-300 °C, WO_3_/Ph-300 °C and WO_3_/PEA-300 °C films, respectively, as listed in Table 1. The much higher coloration efficiency values of the modified WO_3_ films than that of WO_3_-300 °C film can be ascribed to their more efficient Li^+^ ion diffusion and charge transfer in the nanocrystal-embedded amorphous structure. In general, high coloration efficiency provides a large optical contrast with a small charge insertion or extraction, which is beneficial to improving the cycle life of the EC materials, just as our previous work proved that the WO_3_/DA-300 °C film displays excellent stability up to 35,000 cycles [23]. All the modified films exhibit the unique nanocrystal-embedded amorphous structure as shown in TEM images, which consequently help to greatly improve cycling stability of all the modified films.

The switching times, namely coloration and bleaching times, mean the times required for a 90% change in the transmittance contrast during the coloring/bleaching processes, which have been measured from switching curves of the films in Figure 7G and can be seen in Table 1. The switching times for the WO_3_/DA-300 °C film are 10 s for bleaching and 15 s for coloration, while the switching times for the modified WO_3_ film are almost all less than them, indicating that using the additives can accelerate the dynamic response of WO_3_ films. The reason is that the modified WO_3_ films with nanocrystal-embedded amorphous structure possess a much shorter diffusion length of ions due to a larger amorphous region and easier access of a larger amount of Li^+^ ions than the WO_3_-300 °C film with a polycrystalline structure, which can consequently speed up the ion diffusion and charge transfer. Of all the films, the WO_3_/DA-300 °C film shows the shortest switching time (3 s for bleaching and 7 s for coloration), because it possesses the smallest nanocrystals uniformly distributed in an amorphous WO_3_ matrix which may act as ion transport channels and increase reactive active sites during EC process, which is beneficial for the diffusion of the electrolyte into the nanocrystals and the speeding up of the switching response. For most films, the bleaching times are less than the coloration times, showing that the double extraction of cations and electrons from the films is easier and faster than injection into the films.

As we all know, the structure and morphology of WO_3_ films greatly affects EC performance, and amorphous and crystalline WO_3_ films exhibit quite different EC performances from each other [39]. In this study, the introduction of the additives into the precursor PTA sol and the application of an appropriate annealing process promote the formation of nanocrystal-embedded amorphous structure having the merits of both amorphous and crystalline WO_3_ films. All the modified films possess higher EC properties than the pure WO_3_ polycrystalline film, roughly in descending order: the WO_3_/DA-300 °C, WO_3_/CA-300 °C, WO_3_/TA-300 °C, WO_3_/Ph-300 °C and WO_3_/PEA-300 °C films. The strong complexation ability (with W atoms) of DA and CA bidentate additives containing catechol hydroxyl groups limits the size of complex colloidal particles and destroys the structure order of particles to a certain extent, resulting in the formation of more uniform and finer nanocrystals surrounded with an amorphous matrix after heat treatment. On the other hand, among them, DA has the best effect in enhancing the EC properties of the films because it also contains NH_3_^+^ in addition to catechol hydroxyl groups, which can be better dispersed in colloid and thus stabilize colloid solution. TA, PEA and Ph, however, are monodentate additives and show slightly poor complexation ability, resulting in the formation of relatively large and uneven nanocrystals. Especially, PEA contains only one amine group without a hydroxyl group, and the film modified by PEA shows relatively poorer EC performance than other modified films.

## 4. Conclusions

In summary, a series of organic small molecules (DA, CA, TA, Ph and PEA) containing different numbers of hydroxyl and amino groups were added to the PTA sols as structure-directing additives, and a variety of modified WO_3_ films were consequently prepared by a simple and low-cost complexation-assisted sol–gel method. Compared with the pure WO_3_ polycrystalline film, all the modified films combine the advantages of nanocrystalline and amorphous phases and show higher EC properties owing to the unique nanocrystal-embedded amorphous structure. It is worth noting that the additives with different numbers and types of functional groups show different interactions with PTA sol, which affect the size, shape and stability of the complex colloidal particles and consequently change the structures and morphologies of the WO_3_ films from a polycrystalline to nanocrystal-embedded amorphous structure with different grain sizes, finally improving the electrochemical and EC properties of the modified WO_3_ films in different degrees. The additives, in order of their interactions with the sol, are DA, CA, TA, Ph and PEA, primarily depending on the number of hydroxyl groups followed by the number of amino group. Of all the additives, DA with catechol hydroxyl and amino groups shows the most positive effect, that is, the WO_3_/DA-300 °C film exhibits the best EC performances.

Furthermore, the study indicates that the modified WO_3_ films with excellent EC properties prepared by a simple and low-cost complexation-assisted sol–gel method show great application potential for EC devices. The effects of more additives with other functional groups on the microstructure, morphology, and thus EC properties of the WO_3_ films should be further studied in the future to reveal the relationship between structure and performance of the WO_3_ films and further improve the complexation-assisted sol–gel method.

## Figures and Tables

**Table 1:** EC properties of the WO_3_-300 °C, WO_3_/DA-300 °C, WO_3_/CA-300 °C, WO_3_/TA-300 °C, WO_3_/Ph-300 °C and WO_3_/PEA-300 °C films at λ_720nm_.

The Films	∆*T* ^1^ (%)	∆*T* ^2^ (%)	*CE* (cm^2^ C^−1^)	Bleaching Time ^2^ (s)	Coloration Time ^2^ (s)
WO_3_-300 °C	51 ± 1	52 ± 1	66 ± 1	10 ± 1	15 ± 1
WO_3_/DA-300 °C	75 ± 1	75 ± 1	90 ± 1	3 ± 1	7 ± 1
WO_3_/CA-300 °C	72 ± 1	71 ± 1	85 ± 1	5 ± 1	10 ± 1
WO_3_/TA-300 °C	66 ± 1	66 ± 1	81 ± 1	6 ± 1	13 ± 1
WO_3_/Ph-300 °C	64 ± 1	63 ± 1	77 ± 1	9 ± 1	9 ± 1
WO_3_/PEA-300 °C	56 ± 1	55 ± 1	70 ± 1	9 ± 1	15 ± 1

^1^ Obtained from UV-Vis transmittance spectra in Figure 7A–F; ^2^ Obtained from switching curves in Figure 7G.

## Data Availability

All data are available from the corresponding author.

## Supplementary Materials

The following supporting information can be downloaded at: https://www.mdpi.com/article/10.3390/ma16072681/s1, Figure S1: FESEM images of (A) WO_3_-300 °C, (B) WO_3_/DA-300 °C, (C) WO_3_/CA-300 °C, (D) WO_3_/TA-300 °C, (E) WO_3_/Ph-300 °C, and (F) WO_3_/PEA-300 °C films.
