# Supplementary material for: Effects of Additives on Electrochromic Properties of Nanocrystalline Tungsten Oxide Films Prepared by Complexation-Assisted Sol–Gel Method
Source: Materials (Basel). 2023 Mar 28;16(7):2681. doi: 10.3390/ma16072681 (PMC10096167; doi:10.3390/ma16072681)
Supplement: Supplementary file 1 [file materials-16-02681-s001.zip › materials-2293876-supplementary.pdf]

Supporting Information:

Effects of Additives on Electrochromic Properties of Nanocrystalline Tungsten Oxide Films Prepared by Complexation-Assisted Sol–Gel Method

Dan Zhou *, Zhibo Tong, Hongmei Xie, Jiaotong Sun and Fenggui Chen

Chongqing Key Laboratory of Extraordinary Bond Engineering and Advanced Materials Technology, College of Materials Science and Engineering, Yangtze Normal University, Chongqing 408100, China

* Correspondence: zhoudan@yznu.edu.cn; Tel.: +86-023-72791828

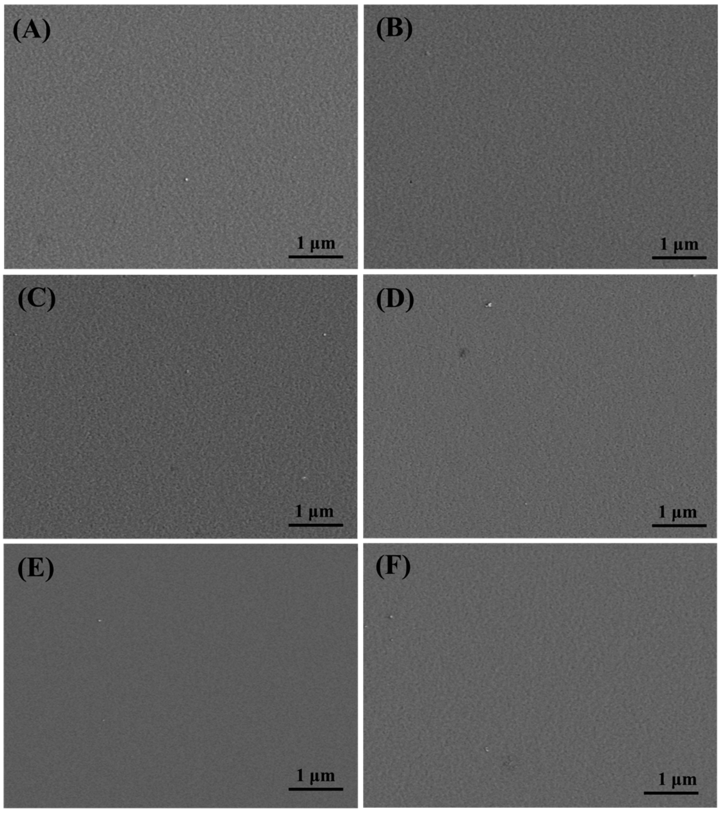

Figure S1. FESEM images of (A) WO_3-300 °C, (B) WO_3/DA-300 °C, (C) WO_3/CA-300 °C, (D) WO_3/TA-300 °C, (E) WO_3/Ph-300 °C, and (F) WO_3/PEA-300 °C films.
